# The Onco-Nephrology Field: The Role of Personalized Chemotherapy to Prevent Kidney Damage

**DOI:** 10.3390/cancers15082254

**Published:** 2023-04-12

**Authors:** Annalisa Noce, Giulia Marrone, Manuela Di Lauro, Anna Paola Mitterhofer, Maria Josè Ceravolo, Nicola Di Daniele, Guglielmo Manenti, Antonino De Lorenzo

**Affiliations:** 1 Department of Systems Medicine, University of Rome Tor Vergata, 00133 Rome, Italy; 2 Nephrology and Dialysis Unit, Policlinico Tor Vergata, 00133 Rome, Italy; 3 Fondazione Leonardo per le Scienze Mediche Onlus, Policlinico Abano, 35031 Abano Terme (PD), Italy; 4 Department of Diagnostic Imaging and Interventional Radiology, University of Rome Tor Vergata, 00133 Rome, Italy; 5 Section of Clinical Nutrition and Nutrigenomic, Department of Biomedicine and Prevention, University of Rome Tor Vergata, 00133 Rome, Italy

**Keywords:** acute kidney injury, chemotherapeutic drugs, chronic kidney disease, renal insufficiency, predictive algorithm

## Abstract

**Simple Summary:**

The onco-nephrology field: the interaction between cancer and kidney disease emphasizes the nephrology–oncology connection. This narrative review focuses on several aspects of this association. First of all, the availability of more effective chemotherapeutic agents, including stem cell therapies and biological drugs, has enhanced the survival of cancer patients. These therapeutic advances ameliorate the outcomes of cancer patients; nevertheless, they could induce secondary effects on renal function. As a consequence, some cancer survivors develop chronic kidney disease (CKD). On the other hand, the coexistence of CKD with cancer reduces the likelihood that cancer patients receive either the optimal anticancer therapy or the supportive care. Nephrologists should be aware of it, starting from an early cancer diagnosis; thus, it is important to set up nephroprotective supportive strategies to avoid the onset of acute kidney injury or the CKD worsening.

**Abstract:**

In recent years, the onco-nephrology field has acquired a relevant role in internal medicine due to the growing number of cases of renal dysfunction that have been observed in cancer patients. This clinical complication can be induced by the tumor itself (for example, due to obstructive phenomena affecting the excretory tract or by neoplastic dissemination) or by chemotherapy, as it is potentially nephrotoxic. Kidney damage can manifest as acute kidney injury or represent a worsening of pre-existing chronic kidney disease. In cancer patients, physicians should try to set preventive strategies to safeguard the renal function, avoiding the concomitant use of nephrotoxic drugs, personalizing the dose of chemotherapy according to the glomerular filtration rate (GFR) and using an appropriate hydration therapy in combination with nephroprotective compounds. To prevent renal dysfunction, a new possible tool useful in the field of onco-nephrology would be the development of a personalized algorithm for the patient based on body composition parameters, gender, nutritional status, GFR and genetic polymorphisms.

## 1. Introduction

In recent years, an increase in renal dysfunction in neoplastic patients related to cancer itself or to chemotherapy has been described [1,2,3]. Therefore, “onco-nephrology” has acquired the characteristics of a new and emerging medicine field. In fact, the prevalence of renal insufficiency (RI) is high in neoplastic patients, especially in those with solid tumors [4]. The first study that examined the RI prevalence in cancer patients was the “IRMA-Renal Insufficiency and Anticancer Medications”-1. This study analyzed a cohort of patients with solid tumors, not on dialysis, highlighting that 52.9% of 4684 enrolled patients had a glomerular filtration rate (GFR) < 90 mL/min/1.73 m^2^, while the 12.0% of this population had a GFR < 60 mL/min/1.73 m^2^ [5,6].

One year after, a following study, called the IRMA-2 study, was conducted on 4945 patients affected by different cancers [6]. IRMA-2 study consisted of two phases: (i) the first phase was a cross-sectional study (like the IRMA-1 study), (ii) the second phase was characterized by a two-year follow-up. In detail, in the IRMA-2 study, the first phase showed that 50.2% of patients had a GFR < 90 mL/min/1.73 m^2^, and 11.9% had a GFR < 60 mL/min/1.73 m^2^. In the second phase, the authors evaluated the impact of cancer on renal function in the time, demonstrating that chemotherapeutic drugs can induce RI if their dose is not adjusted for the GFR.

**Figure 1:**
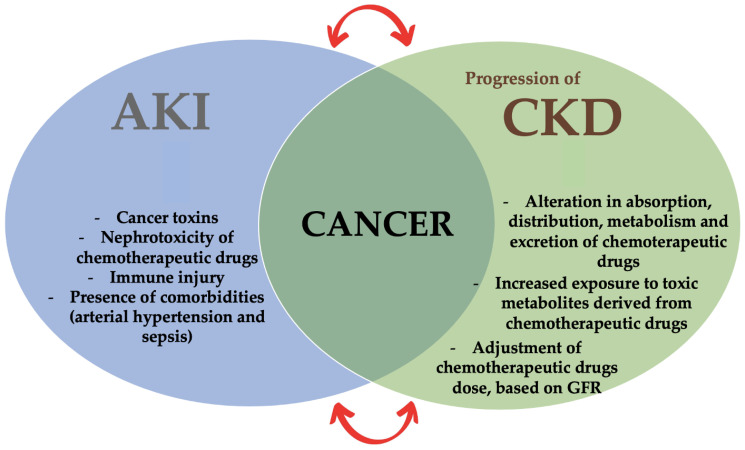
The new medicine field of onco-nephrology.

In cancer patients, both acute kidney injury (AKI) and a progression of chronic kidney disease (CKD) can be observed [7,8]. In fact, in CKD patients, the chemotherapeutic drugs should be tailored based on the GFR in order to avoid the worsening of the residual renal function and to limit the side effects related to chemotherapeutic drugs overdose and, thus, to their toxicity (Figure 1).

Regarding AKI, its severity, degree and incidence are related to the type of cancer, to the chemotherapy used and to the presence of comorbidities that may represent risk factors for RI development [9]. The main risk factors related to the AKI onset in neoplastic patients are the (i) metabolic alterations associated with cancer (such as tumor lysis syndrome-TLS, uric acid nephropathy and hypercalcemia), (ii) the hematopoietic stem-cell transplantation (HSCT) that is often complicated by AKI, (iii) the use of nephrotoxic chemotherapeutic agents and (iv) tissue deposits of paraproteins [10].

An interesting study conducted by Christiansen et al. [11], evaluated the incidence of AKI in a cohort of Danish cancer patients. These patients underwent a five-year follow-up in order to detect the possible presence of AKI, defined according to Risk, Injury, Failure, Loss, and End-Stage Kidney (RIFLE) criteria [12,13]. The one-year risk of developing AKI was 17.5%, while the five-year risk was 27.0%. In detail, a higher risk was observed in patients with kidney cancer (44.0%), liver cancer (33.0%) or multiple myeloma (MM) (31.8%). 

Another epidemiological study was conducted on Canadian patients that received a recent diagnosis of cancer. In this population, the cumulative incidence of AKI was 9.3%. In particular, malignancies inducing a greater risk of AKI at five-year were MM (26.0%), bladder cancer (19.0%) and leukemia (15.4%). Additional AKI risk factors were diabetes mellitus, pre-existing CKD, more advanced cancer stages, age and use of drugs, such as renin–angiotensin–aldosterone system (RAAS) inhibitors and diuretics [14,15]. Moreover, AKI was present in 50–60% of intensive care patients, 20% of whom were affected by a concomitant malignancy [16].

In cancer patients, AKI is correlated with an unfavorable outcome [17]; therefore, it is relevant in the onco-nephrology field to prevent AKI episodes, managing these patients with a multidisciplinary team, possibly avoiding the nephrotoxic chemotherapeutic agents and analyzing the risk and protective factors of AKI [18].

Regarding CKD, there seems to be a bidirectional relationship between CKD itself and cancer [19]. In fact, CKD patients have a higher incidence of cancer compared to the general population, and this phenomenon could be explained by the presence of (i) the low-grade chronic inflammation [20,21,22,23], (ii) the overload of the carcinogenic compounds, (iii) the alteration of DNA repair mechanisms, (iv) the oxidative stress [24] and (v) the gut microbiota dysbiosis [25,26,27,28]. At the same time, cancer patients have a higher risk of developing a concomitant RI. Therefore, there is a significant CKD impact on cancer incidence and on cancer-related mortality.

To corroborate the association between CKD and cancer, a cohort study conducted in the United Kingdom demonstrated that malignancies are one of the main causes of non-cardiovascular death in patients with advanced CKD stages, reaching 15% of all-causes of death in this population [29].

To confirm the second relation, namely the association between cancer and CKD, an observational retrospective cohort study evaluated the prevalence of CKD advanced stages in cancer patients. The authors showed that 12.27% of cancer patients, after one year of follow-up, were affected by CKD stage III-V and this percentage increased up to 13.42% in the two-year follow-up. A higher risk of developing advanced CKD was observed in kidney cancer patients (50%), urinary-tract cancer patients (33.6%) and pancreatic cancer patients (19.6%). On the contrary, a lower risk of developing advanced CKD was observed in colorectal cancer patients (5.3%) and in brain tumor patients (2.5%) [30]. The aim of this narrative review is to evaluate the possible bidirectional correlation between cancer and renal function, through the analyses of the main nephrotoxicity causes and mechanisms induced by chemotherapeutic drugs and by hematopoietic stem cell transplantation. Another purpose of this review is to investigate the most relevant side effects of chemotherapy.

## 2. Methods

The authors conducted literature research using four online databases (PubMed, Web of Science, Cochrane Library and Scopus). Keywords used in searches included “acute kidney injury”, “chemotherapeutic drugs”, “chronic kidney disease” and “renal insufficiency”. Inclusion criteria were studies on the kidney toxic effects of chemotherapeutic drugs both in CKD and in normal renal function cancer patients. Moreover, some articles were excluded because they were not in English language and/or the chemotherapeutic drugs nephrotoxicity was not investigated.

## 3. The Relationship between Glomerular Disease and Cancer

Albuminuria represents an early biomarker of kidney dysfunction, and its presence is related to increased all-causes mortality [31,32]. Moreover, the rise of this biomarker is indicative not only of endothelial dysfunction, as several studies have highlighted its increase also in some types of cancer (such as lung, kidney, breast, colon/rectal and non-Hodgkin’s lymphoma) [33,34,35,36]. This data were confirmed by an interesting study conducted on 5425 subjects without diabetes mellitus or without previous history of neoplasia that examined the possible association between albuminuria and cancer incidence (Table 1). This study showed that an enhanced urinary albumin-to-creatinine ratio (ACR) is directly correlated with cancer incidence [37]. In fact, an elevated ACR is associated with graft versus host disease (GVHD), bacteremia, arterial hypertension (AH), and in CKD patients, with progression of renal dysfunction. On the contrary, this biomarker is not predictive of AKI [38].

The degree of albuminuria seems to reflect the severity of the neoplastic pathology. In fact, it has been shown that patients with metastases or more extensive tumor mass had higher albuminuria levels [35,39]. Elevated albuminuria is associated with an increased cancer incidence, even after an adjustment for the traditional risk factors (such as gender, age, smoking, and body mass index—BMI).

Sometimes, glomerular pathologies can be a paraneoplastic manifestation, and, in particular, membranous nephropathy (MN) is the most frequent glomerular disease associated with cancer [40]. In the 1960s, the link between MN and cancer was described for the first time [41], and subsequently, this association has been repeatedly reported in textbooks [42]. A study by Lefaucheur et al. demonstrated, in both sexes, a higher cancer incidence in MN patients compared to the general population [43]. In detail, the cancer incidence increased in relation to age and to the number of inflammatory cells infiltrating the glomeruli, evaluated through the biopsy. The best cut-off parameter to distinguish cases of cancer-related MN was a number of inflammatory cells infiltrating the glomeruli equal to or greater than eight. This criterion allows a diagnosis of cancer-related MN with a specificity of 75% and a sensitivity of 92% [43].

Furthermore, in the differential diagnosis with the primary MN, it is useful to consider that subepithelial deposits of IgG4, detected by immunofluorescence, are more frequent in idiopathic MN, while those IgG1 and IgG2 are often present in cancer-associated MN [44]. However, many patients with malignancies and proteinuria do not undergo renal biopsy.

About 80% of MN cases have no apparent secondary causes, resulting in their classification as “idiopathic” or “primary” forms [45].

The identification of autoantibodies associated with primary MN began with the discovery of anti-phospholipase A2 receptor (Anti-PLA2R) antibodies, namely the antibodies to podocyte transmembrane glycoprotein M-type phospholipase A2 receptor, in 2009 and of anti-thrombospondin type-1 domain-containing 7A (THSDA7A) antibodies, namely the antibodies against thrombospondin type 1 domain-containing 7A, in 2014 [46]. Based on the current classification, MN in the presence of active cancer is diagnosed as a secondary form and should be negative for anti-PLA2R autoantibodies. Conversely, patients affected by MN associated with positivity for anti-PLA2R antibodies do not require the assessment for secondary causes [47].

However, in 2017 Radice et al. detected anti-PLA2R autoantibodies in 70% of primary MN patients and 28% of secondary MN patients. Whether these cases represented a true secondary MN or even a primary MN associated with concomitant secondary disease is not known. The authors concluded that the anti-PLA2R positivity in a patient with MN should not be a sufficient condition for abstaining from the research of a secondary cause, especially in patients with risk factors for malignancy [48].

Moreover, recent studies have shown that anti-THSD7A antibodies may be associated with cancer-related MN [48]. These findings highlighted the importance of age-appropriate cancer screening, even in patients with positive anti-PLA2R autoantibodies and with presumed primary MN [49].

The main clinical criteria for defining the causal relationship between MN and cancer should include the following:

- the simultaneous or close diagnosis of both the malignancy and the MN;
- the remission of proteinuria in presence of successful cancer treatment and its recurrence in case of the neoplasia relapse [50].

Nonetheless, the treatment should be focused on the fact that patients affected by cancer-associated MN are characterized by a worse prognosis compared to idiopathic MN patients [51].

Moreover, other glomerulopathies have been related to malignancies. In particular, minimal change glomerulonephritis and focal segmental glomerulosclerosis have been frequently associated with solid tumors like lung cancer, colorectal cancer, renal cell carcinoma (RCC) and thymoma, while more rarely with ovarian, breast, bladder and pancreatic cancers [45].

Rapidly progressive glomerulonephritis (RPG) or crescentic glomerulonephritis, as well as membranoproliferative glomerulonephritis (MPG), were reported in association with RCC, lung and gastric cancer [52].

MPG is also observed in hematological neoplastic pathologies [53], as in the case of proliferative glomerulonephritis with monoclonal immunoglobulin deposits (PGNMID). PGNMID is a form of monoclonal gammopathy of renal significance (MGRS), namely a group of disorders that, by definition, do not meet the diagnostic criteria for MM or lymphoproliferative disease. In PGNMID, a monoclonal immunoglobulin secreted by a nonmalignant B-cell or a plasma cell clone causes renal dysfunction [2].

Finally, immunoglobulin A nephropathy is associated with oral mucosa and nasopharyngeal/respiratory tract cancer, colorectal neoplasia, RCC and cutaneous T-cell lymphoma [54,55].

**Table 1:** Main studies on the relationship between glomerular disease and cancer.

Type of the Study	Reference	Year	Methods	Main Findings	Conclusions
*Human* *study*	Pedersen et al. [39]	1996	Evaluation of the prevalence and of the proteinuria prognostic significance in patients with lung cancer.	The presence of proteinuria was significantly more frequent in patients with lung cancer compared to controls. Patients with malignancies and proteinuria had significantly poorer survival than patients with normal urinary protein excretion.	Increased urinary protein excretion may reflect subclinical renal damage related to cancer, and it may also be an independent predictor of poor survival.
	Pedersen et al. [33]	1998	Estimation of the prevalence and the prognostic significance of microalbuminuria in patients with lung cancer.	Increased prevalence of microalbuminuria in patients with lung cancer.	A significant association between microalbuminuria and poor outcome in malignancies.
	Pedersen et al. [35]	2000	Analysis of the frequency and the prognostic significance of microalbuminuria in breast cancer.	An advanced stage of breast cancer is associated with a high prevalence of impaired UAE.	UAE may be a prognostic marker in metastatic breast cancer.
	Pedersen et al. [34]	2003	Evaluation of the association between microalbuminuria and the inflammatory biomarkers in lymphoma patients.	UAE and mediators of inflammation were related to adverse clinical features in patients with non-Hodgkin’s lymphoma.	Direct correlation between microalbuminuria and proinflammatory cytokines in malignancy.
	Lefaucheur et al. [43]	2006	Analysis of the incidence and the characteristics of cancer-associated MN.	Age, smoking and the presence of glomerular leukocytic infiltrates strongly affect the likelihood of malignancy in MN patients.	Epidemiologic evidence of an increased risk of cancer in patients with MN.
	Bjorneklett et al. [50]	2007	Comparison of long-term cancer risk between patients with MN and general population.	An increased long-term risk of developing cancer is observed after the diagnosis of MN.	Patients with cancer and MN had a greater mortality rate than those without cancer.
	Jorgensen et al. [37]	2008	Evaluation of the association between elevated ACR and cancer incidence.	Albuminuria was associated with an enhanced cancer incidence in patients without a history of diabetes mellitus, macroalbuminuria or a previous cancer.	Increased ACR is directly correlated with an enhanced cancer incidence.
	Hingorani et al. [38]	2008	Estimation of the prevalence of albuminuria in patients receiving HSCT.	Elevated ACR was associated with GVHD, bacteremia, AH and, in CKD patients, progression of renal dysfunction. On the contrary, this biomarker was not predictive of AKI.	Kidney injury after HSCT was not always clearly manifested by the changes in sCr and affected long-term outcomes.
	Qin et al. [47]	2011	Estimation of the prevalence of autoantibodies against PLA2R in idiopathic MN.	Presence of anti-PLA2R in idiopatic MN patients was associated with the disease severity.	Anti-PLA2R is a sensitive biomarker for idiopathic MN.
	Qu et al. [44]	2012	Comparison of the IgG subclass of immune complex deposition, clinical and pathological data between patients with cancer-related MN and idiopathic MN.	Subepithelial deposits of IgG4 were more frequent in idiopathic MN, while those of IgG1 and IgG2 were often present in cancer-associated MN.	Absence of glomerular IgG4 deposition, together with older age, severe hypoalbuminemia and high serum CRP level, could be useful clues to differentiate cancer-related MN from idiopatic MN.
	Radice et al. [48]	2018	Comparison of the prevalence of anti-PLA2R antibodies between patients with idiopatic MN and various control groups, including secondary MN.	Positivity of anti-PLA2R autoantibodies in 70% of primary MN patients and in 28% of secondary MN patients.	Anti-PLA2R positivity in MN should not be a sufficient condition for abstaining from the research of a secondary cause, especially in patients with risk factors for malignancy.

Abbreviations: ACR, albumin-to-creatinine ratio; AKI, acute kidney injury; anti-PLA2R, anti-phospholipase A2 receptor; CKD, chronic kidney disease; CRP, C-reactive protein; GVHD, graft versus host disease; HSCT, hematopoietic stem-cell transplantation; MN, membranous nephropathy; UAE, urinary albumine excretion.

## 4. Chemotherapeutic Drugs and Renal Insufficiency

AKI, with a prerenal, intrinsic or postrenal genesis, is the most common nephrological manifestation in cancer patients [56]. The development of AKI in these patients represents a noteworthy event that increases their morbidity and mortality [10]. Furthermore, AKI can alter the bioavailability and/or the safety profile of many chemotherapies. It can enhance the risk of toxic effects or it can lead to suboptimal treatments due to the need to reduce the dose or to use alternative therapeutic schemes [57].

Although nephrotoxicity is a major side effect of many chemotherapeutic drugs, not all patients treated with these agents develop AKI [58].

The causes of kidney failure are related to various factors, including the type of neoplastic disorder, the pharmacological treatment [59] or patient-specific parameters [60]. In particular, in older patients with reduced GFR, the nephrotoxicity is more common [61].

In addition, sex can impact on side effects related to pharmacotherapy, mainly through variables such as body weight and/or body composition. On average, men have a higher BMI and a wider body surface area compared to women. These differences in body size result in larger distribution volumes and faster clearance of most drugs. A greater amount of body fat may increase distribution volumes for lipophilic therapeutic agents [62].

Chemotherapy can cause AKI with several mechanisms. In addition to the conventional chemotherapeutic agents responsible for acute tubular toxicity, acute tubulointerstitial nephritis (ATIN) and glomerular injuries, the new lines of treatment, including immunotherapies and targeted cancer therapies, have increased the occurrence of kidney immune-mediated injury (Table 2) [2].

Among conventional chemotherapeutic drugs, cisplatin deserves a special mention. This drug is the most commonly used in cancer treatment and it can induce nephrotoxic side effects by tubular damage [63]. In detail, the nephrotoxic effect is related to its dosage, and the kidney injury induced by cisplatin seems to be reversible after its discontinuation [64]. The tubular damage seems to be mediated by a membrane transported, called organic cation transporter 2 (*OCT*2). OCT2 exerts its action through cisplatin transport into renal tubular epithelial cells [65]. Consequently, OCT2 is responsible for the cellular uptake of cisplatin and, thus, for its intracellular accumulation. In animal models, the deletion of *Oct1* and *Oct2* genes alters the urinary cisplatin excretion without affecting its plasma levels. Furthermore, carrier patients of the *Oct2* polymorphism seem to have a lower risk of developing cisplatin nephrotoxicity [66]. AKI, present in 20–30% of patients’ cisplatin exposed, is usually non-oliguric. Moreover, urinalysis may detect glycosuria and a low-grade proteinuria. AKI-related cisplatin may also be associated with tubulopathies, such as Fanconi-like syndrome, hypomagnesemia, salt-loosing syndrome and distal renal tubular acidosis [67]. Tubular dysfunction is characterized by electrolyte disorders such as hyponatremia, hypokalemia and hypomagnesemia. Other reported manifestations are thrombotic microangiopathy (TMA) and anemia due to the deficit of erythropoietin [68].

The novel chemotherapeutic agents, namely vascular endothelial grow factor (VEGF) inhibitors (such as bevacizumab and sunitinib) or antimetabolites (such as gemcitabine) might cause kidney injury due to the development of TMA.

In particular, VEGF-target treatment can induce proteinuria and AH. Therefore, kidney side effects related to the use of this pharmacological treatment are due to the production of VEGF by the renal visceral epithelial cells [69]. Moreover, VEGF binds receptors sited on glomerular podocytes, mesangium and peritubular capillaries. About 80% of cancer patients treated with this chemotherapeutic drug develop AH [70]. Therefore the blood pressure monitoring plays a pivotal role in the clinical management of these patients because it represents an early clinical biomarker of renal dysfunction.

Another class of novel chemotherapeutic drugs is the immune checkpoint inhibitors (ICPIs) that might induce interstitial nephritis [63]. In detail, ICPIs, such as nivolumab and pembrolizumab, stimulate T-cells to kill cancer cells, counteracting the bind of dendritic cells with cytotoxic T-lymphocyte-associated protein (CTLA)-4 and the bind of tumor antigen ligand with the programmed death (PD)-1 receptors [71]. The incidence of renal immune-related damage induced by ICPIs ranges from 2% to 5%. In fact, these drugs not only can cause acute interstitial nephritis but also glomerular disease. The most frequently reported glomerular lesions related to the ICPIs treatment are the pauci-immune GN, the podocytopathies and the C3 GN [72].

Another innovative use of immunotherapy to treat several forms of cancers, named CAR T-cell therapy, is based on the collection of patients’ T cells in order to genetically modify them and to permit the expression of antigen receptors, normally not present [73]. The result of this new technique is the creation of a chimeric molecule characterized by T cells with their specific antibodies [74]. This therapy is associated with a storm cytokine and with an AKI induced by reduced renal perfusion related to hypotension [75].

Additionally, in cancer patients, non-steroidal anti-inflammatory drugs (NSAIDs), organo-iodinated contrast and other potentially nephrotoxic therapeutic agents (i.e., aminoglycosides, vancomycin or acyclovir), whenever possible, should be avoided to reduce the AKI risk [76].

**Table 2:** Main studies on chemotherapeutic drugs and renal insufficiency.

Type of the Study	Reference	Year	Methods	Main Findings	Conclusions
*Human* *study*	Stewart et al. [68]	1997	Evaluation of factors affecting cisplatin nephrotoxicity.	Negative association between cisplatin nephrotoxicity and serum albumin levels, potassium, body surface area, and the use of vinca—alcaloid. Positive association with use of metoclopramide.	Serum albumin, metoclopramide and phenytoin affected the nephrotoxicity by altering cisplatin uptake into the kidney.
	Robinson et al. [70]	2010	Time evaluation for AH and proteinuria onset in patients receiving cediranib (a VEGF receptor inhibitor).	Cediranib induced a rapid but variable rise in blood pressure and of proteinuria.	Understanding the mechanisms that regulate VEGF inhibitor-induced will permit to manage vascular tone and endothelial health.
*Animal* *study*	Ciarimboli et al. [65]	2005	Investigation of the interaction of cisplatin with hOCT2 in kidney or hOCT1 in liver through a florescent cation.	Uptake of cisplatin is mediated by hOCT2 in renal proximal tubules, explaining its organ-specific toxicity.	A combined administration of cis-platin with other substrates that compete for hOCT2 offers an effective therapeutic option to decrease its nephrotoxicity.
	Filipski et al. [66]	2009	Comparison of cisplatin nephrotoxicity in groups of OCT1/OCT2-deficient mice.	*Oct2* polymorphism seems to give a lower risk of developing cisplatin nephrotoxicity.	Critical relevance of *Oct2* in the clinical and therapeutic management of cisplatin-treated patients.

Abbreviations: OCT, organic cation transporter; VEGF, vascular endothelial grow factor.

## 5. Kidney Dysfunction after Hematopoietic Stem Cell Transplantation

HSCT represents a life-saving treatment for several neoplastic patients [77]. Either AKI or CKD are very common in HSCT patients, and their presence is responsible for the increased mortality [78].

An important study conducted by Zager RA et al. examined 272 adult patients after myeloablative transplant (of which 89% had received an allogenic transplant and 11% had received an autologous transplant) (Table 3). The authors concluded that 55% of enrolled patients developed AKI after HSCT, and half of these requested renal replacement therapy [79]. These data were confirmed by the following studies [80,81].

The mechanisms that directly induce kidney damage after transplantation can be vascular, glomerular and/or tubulointerstitial.

The risk factors of renal injury in HSCT patients are the type of chemotherapeutic drugs, the presence of sepsis, the TMA, the radiotherapy, the type of transplant performed, etc.

AKI onset is more frequent during the first month of HSCT [82,83]. In fact, in this period, the patients are more exposed to developing AKI, due to the more susceptibility to sepsis, to the high dose of nephrotoxic drugs administered in this phase [84] and to the hepatic sinusoidal obstructive syndrome. In fact, sepsis, often present in immunocompromised patients undergoing HSCT, can be complicated by AKI. The latter can be induced by renal hypoperfusion, secondary to systemic vasoconstriction, and by the intrarenal endothelial dysfunction associated with capillary thrombosis. Moreover, nephrotoxic drugs, such as vancomycin and aminoglycosides, often administered in this condition, may furtherly contribute to the development of sepsis-related AKI. Patients in treatment with aminoglycosides, more exposed to developing AKI, are those previously affected by CKD, liver disease and dehydration state [85,86].

The hepatic sinusoidal obstructive syndrome after HSCT can also provoke AKI onset. This syndrome is caused by sinusoidal obstruction, secondary to damage of sinusoidal endothelial cells and hepatocytes. It can be considered another form of hepatorenal syndrome and its most frequent signs and symptoms are jaundice, painful hepatomegaly and overhydration [87,88].

Another cause of AKI in HSCT patients is the marrow infusion syndrome (MIS) that is induced by stem cell treatment before HSCT. In fact, in order to preserve the steam cells, it is necessary to treat them with cryoprotective substances, such as dimethyl sulfoxide (DMSO). Such treatment can induce lysis of red blood cells (RBCs). Therefore, the infusion of RBCs during transplantation can cause vomiting, fever, nephropathy and blood pressure fluctuations, and in these cases, AKI might be caused by renal vasoconstriction and hemoglobin-induced cytotoxicity [89].

The prevention and treatment strategies of HSCT-associated AKI include: (i) the prevention and the monitoring, (ii) the general clinical treatment and (iii) the specific therapeutic treatment.

The former strategy encloses the achievement of a hydration optimal state, the careful use and/or the dose adjustment of nephrotoxic drugs and organo-iodinated contrast medium and the frequent and scheduled monitoring of renal function.

Further strategies include the suspension of nephrotoxic drugs in the event of the AKI onset, and the clinical and pharmacological treatment of the septic state, if present. The specific therapeutic treatment is characterized by the use of steroids, in the case of marrow infusion syndrome, by the use of albumin, terlipressin and defibrotide, in the case of a hepatic sinusoidal obstructive syndrome, and by the treatment of the hypertensive state and the suspension of calcineurin inhibitors, in the case of TMA [77].

In fact, TMA is a possible complication of HSCT. It is characterized by diffuse endothelial damage with consequent formation of thrombi in the microcirculation and by ischemic phenomena affecting the kidneys. It is not clear yet, whether TMA represents a complication of transplantation or it is secondary to infectious phenomena, drug toxicity or the GVHD onset [90].

As regards CKD, the incidence of renal dysfunction after HSCT appears to be variable. In fact, several clinical studies report that this condition can range from 0% to 60% in the following 6 months after transplantation [91,92,93]. This variability may be related to the different duration of follow-up, to the use of different definitions of renal dysfunction, and, lastly, to the type of transplant.

Another aspect to evaluate is that the progression of renal disease, which occurs after HSCT, towards ESRD is much faster than that observed in the general population [94]. A prospective cohort study by Hingorani et al. [95], conducted on 432 adult patients undergoing HSCT, evaluated the GFR trend with a median follow-up period of 5.3 years after the transplantation. The authors observed a greater reduction in estimated-GFR (e-GFR) during the first year after the transplantation. Specifically, the mean baseline e-GFR decreased from 98 mL/min/1.73 m^2^ to 78 mL/min/1.73 m^2^. The authors noticed that after the initial decline, e-GFR was stable and its further decreases were associated with an increased risk of mortality.

The risk factors related to the development of CKD after HSCT include old age, a pre-existing presence of reduced GFR, female gender, pharmacological therapy based on calcineurin inhibitors, the presence of glomerular pathologies, the TMA and the GVHD [96].

Another risk factor of CKD after transplantation is represented by radiation therapy [97].

Furthermore, the urinary ACR seems to be a valid diagnostic tool for monitoring HSCT patients, as its increase represents a risk factor for adverse clinical events.

Currently, knowledge regarding the impact of allogenic HSCT on renal function in the pediatric population is limited. It is well known that the previous use of nephrotoxic chemotherapeutic drugs, the co-presence of AH or infectious, in association with myeloablative or prophylactic therapy against GVHD, increases the risk of developing AKI in the pediatric population [98,99]. Moreover, 1/3 of children with post-HSCT AKI may develop CKD [100].

A recent and interesting retrospective study by Musiał et al. [101,102] evaluated renal function in children undergoing allogenic-HSCT, analyzing the possible differences between patients transplanted for oncological causes compared to those transplanted for non-oncological causes. The authors concluded that children undergoing HSCT, both for oncological indications and for those non-oncological, have the same risk of developing AKI, as defined by Kidney Disease: Improving Global Outcome (KDIGO) [103] and pRIFLE criteria [104]. Moreover, oncological patients seem to be more predisposed to maintain renal damage over time. Confirming this study’s importance and excellent methodology, only the pRIFLE criteria seem to be suitable in the pediatric population. In fact, the acute kidney injury network (AKIN) and KDIGO [103] classifications examine the changes in serum creatinine concentration; on the contrary, the pRIFLE criteria are based on the reduction of e-GFR and urinary output [105]. In addition, the pRIFLE criteria consider the possible reversibility of the kidney damage and the AKI progression toward CKD. It is widely known that serum creatinine is a non-timely biomarker of renal dysfunction and it is also influenced by several factors, such as gender, age, muscle mass and body composition of the patient.

**Table 3:** Main studies on kidney dysfunction after hematopoietic stem cell transplantation.

Type of the Study	Reference	Year	Methods	Main Findings	Conclusions
*Human* *study*	Zager et al. [79]	1989	Assessment of the incidence and the risk factors of AKI following BMT.	AKI development after BMT was preceded by hepatic dysfunction, overweight, amphotericin B use, septicemia and hypotension, with a grave prognosis.	AKI, with hemodinamic genesis, was a common complication of BMT.
	Parikh et al. [80]	2002	Evaluation of the renal dysfunction in patients undergoing HCT	After HCT, severe nephrotoxicity was associated with sepsis, hepatic toxicity, VOD and lung toxicity	To prevent renal dysfunction, the potential nephroprotective drugs must be started soon after allogenic HCT for a 2- to 3-week period.
	Parikh et al. [81]	2005	Comparison of the prevalence of ARF between myeloablative and nonmyeloablative HCT.	The incidence and the severity of ARF, which occurs after nonmyeloablative HCT, is significantly lower compared with myeloablative HCT.	Nonmyeloablative HCT might decrease ARF frequency, improving outcomes in case of advanced hematologic neoplasia.
	Hingorani et al. [91]	2007	Evaluation of CKD risk factors after HCT.	The occurrence of CKD was rare, often associated with ARF and GVHD.	Prevention of GVHD could reduce the CKD incidence following HCT.
	Pinana et al. [83]	2009	Evaluation of the incidence and the risk factors of ARF after reduced-intensity conditioning Allo-HSCT (Allo-RIC).	ARF was a frequent complication after Allo-RIC, with a negative impact on outcome.	ARF identification risk factors help to avoid exposure to nephrotoxic drugs during the follow-up in high-risk patients.
	Liu et al. [82]	2010	Estimation of the incidence and AKI risk factors following nonmyeloablative HSCT.	AKI was common and associated with incomplete HLA-matched transplant and its complications, with poor long-term outcome.	AKI may be related to a worse outcome.
	Hingorani et al. [95]	2018	Evaluation of the association between changes in e-GFR values and the all-cause mortality after HCT.	Adult HCT recipients had a greater reduction in e-GFR during the first year after the transplantation.	After the initial decline, e-GFR was stable, and its further decreases were associated with an increased risk of mortality.
	Musial et al. [101]	2021	Assessment of renal function in children undergoing allogenic-HSCT due to oncological and non-oncological causes.	Children of both groups demonstrated the same risk of AKI, but oncological patients seem to be more predisposed to sustained renal injury.	The pRIFLE criteria seem to be the only one applicable to the pediatric population.
	Augustynowicz et al. [102]	2021	Evaluation of AKI incidence in children undergoing AlloHSCT.	The AKI incidence in children undergoing alloHSCT It is independent of indication for this procedure, whereas eGFR values seem conditioned by previous chemotherapy in oncological patients The AKI incidence in children undergoing alloHSCT It is independent of indication for this procedure, whereas eGFR values seem conditioned by previous chemotherapy in oncological patients The AKI incidence is independent of the indication for AlloHSCT, whereas e-GFR was afflicted by the previous chemotherapy in oncological patients.	Children undergoing AlloHSCT due to oncological causes had a greater risk of kidney dysfunction within 6 months.

Abbreviations: AKI, acute kidney injury; ARF, acute renal failure; BMT, bone marrow transplantation; CKD, chronic kidney disease; e-GFR, estimated-glomerular filtration rate; GVHD, graft versus host disease; HLA, human leukocyte antigen; HSCT, hematopoietic stem-cell transplantation; VOD, veno-occlusive disease.

## 6. Other Causes of AKI in Cancer Patients

Other possible causes of AKI in cancer patients could be urinary tract obstruction and/or compression, as observed in prostate, urothelial, uterus or ovary tumors or in the presence of metastases [76]. In case of leukemia and lymphoma, cancer cells may infiltrate the kidney; in such cases, AKI is attributable to the destruction of kidney microvascular and tubular structures [106].

In dysproteinaemias, the mechanisms responsible for AKI are the precipitation, the aggregation or the misfolding of the paraprotein in different kidney sites and/or the hypercalcemia [2].

TLS is another common cause of cancer-induced AKI [107] and the underlying mechanisms that lead to TLS are cancer cell death, which can be spontaneous or chemotherapy-induced. Cancer cell death can induce hyperuricemia, hyperkalemia, hyperphosphatemia and hypocalcemia [108]. In the presence of TLS, the AKI onset is due to a combination of inflammatory tubular injury, acute uric acid nephropathy and acute nephrocalcinosis [10].

## 7. Administration of Chemotherapeutic Drugs in CKD Patients

It is essential to evaluate the patient’s GFR when administering the chemotherapeutic drugs. In fact, the RI presence influences their pharmacokinetics. There are four phases of pharmacokinetics for each drug: absorption, distribution, metabolism and elimination/excretion [4]. In CKD patients, an alteration of gut metabolism can be observed, such as the down-regulation of intestinal cytochrome p450, which alters the amount of drug that reaches the systemic circulation [109]. This phenomenon could affect the absorption phase.

The distribution phase is determined by the drug distribution volume, which represents the drug’s ability to diffuse itself once administered. In CKD patients, the distribution volume can be altered due to the variation in the concentration of drug-binding proteins. In fact, in CKD, hypoalbuminemia or a higher serum concentration of α1-glycoprotein can often be observed. Therefore, this impairment of drug-binding proteins causes an increased free fraction of acid drugs and a reduced availability of basic drugs [110]. Before excretion, drugs undergo a metabolic transformation in the liver. The metabolism of biliary-excreted chemotherapeutic drugs and of those metabolized by hepatic cytochrome p450 may be impaired in CKD patients.

In the end, the kidney is heavily involved in the elimination of the drugs, and, as a consequence, kidney damage can lead to an altered excretion of the drugs and their metabolites [111]. The main chemotherapeutic agents, partially excreted through the kidney, are carboplatin, cisplatin, mitomycin, methotrexate, pemetrexed, pentostatin, topotecan, bleomycin, capecitabine and etoposide [112].

For these reasons, patients with RI have a higher risk of developing side effects from overdose compared to cancer patients with normal renal function. Therefore, the dosage of chemotherapeutic drugs in CKD patients should undergo an adjustment, according to the GFR.

Regarding the correct administration dose of chemotherapeutic drugs, it is important to consider that serum creatinine (sCr) levels allow approximately an estimate of the real renal function. In fact, as described above, they seem to be influenced by several factors, such as age, sex, race, muscle mass, nutritional status, meat intake and creatine supplementation [113]. It is important to underline that sCr levels overestimate the renal function. In particular, about 60% of patients with normal sCr levels present an impaired renal function. Indeed, from 5% to 15% of the patients with both estimated creatinine clearance (eCrCl), expressed in milliliters/minute, and eGFR, expressed in milliliters/minute/1.73 m^2^, lower than 60, present sCr values within the normal range [5,8].

Nowadays, to evaluate the correct dose of chemotherapeutic drugs according to real renal function, the Chronic Kidney Disease Epidemiology Collaboration (CKD-EPI) equation should be used. Unfortunately, in the current clinical practice, several administration doses of chemotherapeutic drugs are determined using the Cockcroft–Gault (CG) formula [114]. This formula provides an inaccurate estimate of GFR due to its inability to adequately compensate several factors that can influence the sCr values, as previously described. Further evidence of this inaccurate estimate of GFR using the CG formula is given by the fact that the direct measurement of CrCl, obtained through 24-h urine collection, was reported in the validation study of this formula [115,116,117].

Up to now, validation studies for the formula based on the combination of creatinine and cystatin C serum levels in the cancer population have not been performed yet. Cystatin C cannot be considered an ideal GFR surrogate because its synthesis is influenced by inflammation and cell turnover [118].

On the contrary, the CKD-EPI equation, currently recommended by the National Kidney Foundation—Kidney Disease Outcome Quality Initiative (KDOQI) and the KDIGO guidelines [119], has been validated in cohort studies on cancer patients [120].

In a recent study, Klockl et al. concluded that it is useful to evaluate the renal function with only eGFR (namely omitting the burdensome timed urine collection for CrCl measurement) for patients with solid cancers who have received at least one cycle of cisplatin therapy (Table 4) [121].

However, the debate on the use of CG formula or CKD-EPI formula is still ongoing. Only few studies provide specific recommendations on the preferred method to estimate kidney function in patients with malignancy. The National Comprehensive Cancer Network (NCCN) suggests using CrCl in the elderly and “GFR calculations” in adolescents and young adults [122,123], while the International Society of Geriatric Oncology (SIOG) suggests using the MDRD equation for cancer patients > 65 years [124]. The food and drug administration (FDA) guidelines, on the contrary, recommend the CG formula [125].

In clinical practice, a possible approach should include the evaluation of the concordance between eGFR values, calculated through different formulae, as previously described. For differences < 10 mL/min or <10%, we should use the recommended dose for the drug; at the same time, we should consider the patient’s vulnerability and the drug’s nephrotoxicity profile. In the case of drugs with a narrow therapeutic range, it may be useful to adjust the dose based on the formula that calculates the lower eGFR [125]. Furthermore, repeated assessments of GFR and eGFR should be considered, especially in patients with changes in body mass and composition.

The possibility to directly measure the GFR with an accurate, rapid and reproducible methodology is necessary.

Radionuclide and radiocontrast methods are not typically used in daily clinical decisions, even though they are very accurate. Moreover, these methods are not widely available and they can cause adverse events related to radiations exposure and to the risk of anaphylaxis. In oncology, they can be used to confirm the GFR values obtained through other techniques or to determine the GFR in undefined clinical situations, as for pathological BMI values or no steady-state conditions [126].

In order to personalize medical care, a recent methodology seems to let us to obtain a direct quantitative measurement of the GFR. This method is performed by a transdermal system, using a small light sensor placed on the patient’s skin and a biocompatible fluorescent tracer (relmapirazin, MB-102), removed from the blood exclusively by glomerular filtration. The measured florescence decreases over time; gradually, the tracer is cleared by the kidneys, and at the same time, the software converts the time-dependent fluorescence curve into GFR, thanks to specific algorithms [127]. The measured GFR would be used to adjust the dose of chemotherapeutic drugs in order to obtain the minimal toxicity and the maximal efficacy.

**Table 4:** Main studies on the administration of chemotherapeutic drugs in CKD patients.

Type of the Study	Reference	Year	Methods	Main Findings	Conclusions
*Animal* *study*	Henderson et al. [110]	1992	Comparison between the uptake of 5-Propyl FPA and of the PAH in rat kidney slices.	5-Propyl FPA underwent an active tubular secretion in a similar way to PAH.	5-Propyl FPA inhibited the renal excretion of various drugs, conjugates and other endogenous organic acids.
	Leblond et al. [109]	2002	Evaluation of CRF effects on intestinal cytochrome P450 in rats.	Reduction of creatinine, total intestinal cytochrome P450 activity, CYP1A1 and CYP3A2 expression.	CRF was associated with a decrease in intestinal cytochrome P450 activity secondary to reduced gene expression.
*Human* *study*	Inker et al. [117]	2012	Performance comparison between two equations for the GFR estimation (one using standardized cystatin C alone and the other using cystatin C combined with standardized creatinine).	The combination of serum creatinine with serum cystatin C is more accurate than any other marker alone for the GFR estimation.	eGFR based on serum cystatin C could be used as a confirmatory test for renal function evaluation.
	Janowitz et al. [120]	2017	Evaluation of the most accurate published models and development of a new model to estimate the GFR.	BSA-adjusted CKD-EPI formula is the most accurate published model to estimate the GFR in patients with cancer.	The newly developed model improves GFR estimation, and it may represent a new tool for clinical management.
	Klockl et al. [121]	2020	Evaluation of reliability to determine the eGFR before cisplatin therapy, omitting uCrCl measurement.	GFR estimated by CKD-EPI formula is reliable in patients with solid cancers undergoing cisplatin therapy and who have received at least one cycle of chemotherapy.	GFR allows a correct assessment of kidney function in patients with cancer undergoing cisplatin therapy.

Abbreviations: BSA, body surface area; CKD-EPI, chronic kidney disease epidemiology collaboration; CRF, chronic renal failure; GFR, estimated-glomerular filtration rate; FPA, furanpropanoic acid; PAH, P-Amminoippurate; uCrCl, calculating urine creatinine clearance.

## 8. Most Common Electrolyte Disorders in Malignancy

In cancer patients, electrolyte disorders are frequent due to tubular functional abnormalities related to chemotherapeutic drugs, the TLS and the impaired secretion of antidiuretic hormone (Table 5) [9]. These alterations usually involve sodium, calcium, potassium and magnesium serum levels. In cancer patients, the sodium alteration is the most common electrolyte imbalance detected. In some cases, this electrolyte impairment could be the first sign of an undiagnosed cancer [128]. Hypernatremia seems to be caused by diabetes insipidus related to brain metastasis, anorexia, and, in the most severe cases, to cachexia and gastro-intestinal disorders [129]. Moreover, this electrolyte alteration is ascribable to chemotherapeutic drugs, inducing vomiting and diarrhea [130]. At the same time, hyponatremia should be related to brain and adrenal metastasis, paraneoplastic syndrome, and to chemotherapeutic drugs or to supportive therapy based on diuretics, opioids and antibiotics [131]. Patients with hyponatremia associated with renal salt-loosing syndrome require acute treatment with intravenous (IV) saline solution to restore volume depletion. In the second phase, salt tablets can be administered to prevent symptomatic hypotension, although the patient is euvolemic.

Hypercalcemia is commonly induced by MM or an advanced-stage malignancy.

Instead, the hypokalemia should be caused by gastro-intestinal or renal loses induced by some classes of chemotherapeutic drugs and by the paraneoplastic or ectopic secretion of adrenocorticotropic hormone [132]. On the contrary, hyperkalemia is related to TLS in most cases. 

Lastly, hypomagnesemia is caused by gastrointestinal loses or renal tubular dysfunctions secondary to the chemotherapy [133]. In patients with refractory hypomagnesemia, it could be useful the administration of a potassium-sparing diuretic (i.e., amiloride) [134].

In this context, in cancer patients, it is very important the frequent monitoring of serum electrolytes in order to early diagnose possible disorders and to timely treat them [9]. In fact, the electrolyte imbalance can often be the cause of premature death in these patients.

As previously described, also the cisplatin can induce electrolyte imbalances. In this case, the standard approach to avoid cisplatin-induced nephrotoxicity and the consequent electrolyte alteration is the administration of IV isotonic saline solution in order to stimulate the diuresis. Some authors suggest to add 2 gr of magnesium sulfate and 20 mEq of potassium chloride into the isotonic saline solution. This solution should be administered two-three hours before and two hours after the chemotherapy based on cisplatin. The aim of this protocol is to obtain at least 100 mL/h of urine output [135]. In the case of renal tubular acidosis induced by cisplatin, patients should also receive alkali therapy (i.e., sodium bicarbonate or potassium citrate).

Mannitol may be used to force diuresis, in selected cancer patients, like those treated with high dosages of cisplatin or those affected by AH [135].

Interestingly, amifostine (2-(3-aminopropylamino) ethylsulfanyl phosphonic acid) is a pharmaceutical antidote to cisplatin, approved by the FDA, for reducing cumulative nephrotoxicity due to repeated cisplatin administrations. Amifostine has been recommended at the dose of 910 mg/m^2^, administered IV, 30 min before and 15 min after the chemotherapy. However, during its infusion, it is required patient close monitoring, as it could induce acute hypotension, fatigue and nausea [136,137].

Over time, several other preventive approaches have been proposed to avoid cisplatin-induced nephrotoxicity. These agents included N-acetylcysteine [136], sodium thiosulfate [138], glycine [139], theophylline [140], lithium [141] and cell cycle inhibitors [142].

**Table 5:** Main studies on the electrolyte disorders in malignancy.

Type of the Study	Reference	Year	Methods	Main Findings	Conclusions
*Animal* *study*	Heyman et al. [140]	1993	Evaluation of the effect of glycine infusions on the early renal uptake of cisplatin.	Kidney platinum content was markedly lower in rats who had received glycine infusion compared to control rats who had received saline-infusion.	Glycine infusions reduce early renal accumulation of cisplatin.
*Human* *study*	Wu et al. [138]	2005	Evaluation of NAC chemoprotection in human tumor cell lines.	NAC blocks both the death receptor and the mitochondrial apoptotic pathways induced by cisplatin.	NAC can protect against chemotherapy side effects.
	Benoehr et al. [141]	2005	Assessment of the possible nephroprotective effect of theophylline during cisplatin-based chemotherapy.	Patients who had received theophylline had no GFR deterioration.	Theophylline may prevent AKI induced by cisplatin-based chemotherapy.
	Ingles Garces et al. [128]	2018	Evaluation of the incidence, of the severity and of the prognosis of EAs in phase I clinical trials.	EAs (particularly hypoNa, hypoK, hypoP, hypoMg and hypoCa) are common in cancer patients and may worsen patients’ prognosis.	Careful monitoring and early treatment are proposed to avoid EAs.
	Cheminet et al. [133]	2018	Evaluation of clinical characteristics and of biological abnormalities in patients with extreme hypoMg.	Extreme hypoMg is rare and is frequently associated with severe hypoCa.	Digestive disorders and drugs are the main EAs causes.

Abbreviations: AKI, acute kidney injury; EAs, electrolyte abnormalities; GFR, glomerular filtration rate; NAC, N-acetylcysteine.

## 9. Discussion

From the careful evaluation of the examined clinical studies, related to the onconephrology field in the clinical management of neoplastic patients and/or of patients with suspected neoplasia, it would be advisable to carry out the following indications:

1- In the case of glomerulopathies, it is necessary to exclude the presence of cancer. Therefore, it would be useful to perform an age-related cancer screening in patients with proteinuria.
2- In subjects at high risk for cancer, in case of anti-PLA2R antibodies positivity, the secondary MN should not be totally excluded. Therefore, it would be advisable to carry out also screening for neoplastic pathologies.
3- In cancer patients treated with cisplatin, it would be of notable interest to include in the clinical routine the monitoring of *Oct2* polymorphism, as the subjects carrying such polymorphism have a low risk to develop cisplatin-related AKI. For this reason, they would be the ideal candidates for such chemotherapy. At the same time, those who are at higher risk to develop cisplatin-related AKI should be treated, whenever possible, with another chemotherapeutic drug.
4- In the case of therapy based on the VEGF target, the monitoring of arterial blood pressure is of primary importance, as this parameter often represents an early marker of renal damage.
5- In the case of HSCT, it is important to monitor the renal function to prevent AKI, above all, within the first month. Specific nephroprotective treatments should be developed in addition to the standard clinical approaches.
6- To prevent HSCT-associated AKI, it should be advisable to look for preventive strategies, to perform a careful monitoring of the renal function and of the clinical conditions and to carry out standard and specific clinical treatments.
7- In pediatric patients, who underwent allogeneic HSCT, pRIFLE criteria seem to be the most effective strategy to monitor AKI.
8- The pharmacokinetics of chemotherapeutic drugs in pre-existed CKD patients are affected by impaired renal function. Therefore, the dosage of chemotherapies drugs should be tailored according to the most reliable GFR formula.
9- In order to adjust the dose of the chemotherapeutic drug in the most effective and safest way, it would be advisable to estimate the GFR with the various formulas, evaluating the possible agreement between the results obtained in order to estimate the real renal function.
10- In cancer patients often occur electrolytes imbalances; therefore, the frequent monitoring of serum electrolytes plays a pivotal role in the clinical management of these patients.

## 10. Conclusions

Nephroprotective strategies should be adopted in cancer patients. When the physician sets the chemotherapy, he/she should try to preserve renal function, avoiding as much as possible the use of nephrotoxic drugs and, when they are necessary, adjust their dose in relation to patients’ GFR. Moreover, it is important to avoid the concomitant assumption of several nephrotoxic drugs belonging to different classes (such as NSAIDs, antibiotics and chemotherapeutic drugs). Anyway, it is not always possible, as we deal with patients seriously compromised, which often face infections caused by multiresistant strains. For either CKD patients or those who are at risk of developing AKI, the therapy should be personalized. In particular, we suggest to choose the less nephrotoxic drug among those effective to counteract the infection. Moreover, the physician should evaluate further risk factors either bound to the therapy or to the presence of comorbidities. The clinician should also ensure an optimal state of hydration (for example, to stop diuretic therapy and start a proper infusion therapy) and plan a short-term follow-up of the renal function. Lastly, it is necessary to consider that the antibiotic, antiviral and antifungal therapy, although at risk for this kind of patient, are often life-saving and, consequently, inevitable. Nevertheless, the physician should always evaluate the best therapeutic strategy, working in a multidisciplinary team to estimate the risk-benefit ratio for every administered drug.

As reported by the BIRMA study, a significant number of enrolled patients [8] with RI did not receive the correction, based on the GFR, of the dose of the chemotherapeutic drug. Indeed, we re-affirm the necessity of a multidisciplinary team (including oncologists, nephrologists, hematologists and pharmacologists) to manage cancer patients. In the presence of RI or in patients with a high risk of developing AKI, it should be useful the preventive administration of nephroprotective compounds (such as N-acetylcysteine and 2-melcaptoethanesulphonate), in addition to appropriate hydration.

In order to evaluate the correct drug dose to administrate to the cancer patients, it should be advisable to elaborate, through an artificial intelligence system, an algorithm comprising fundamental parameters, such as body composition, anthropometric measurements, nutritional indices, GFR calculated with CKD-EPI formula, gender and genetic polymorphisms.

## Abbreviations

ACR	Albumin-to-creatinine ratio
AH	Arterial hypertension
AIN	Interstitial nephritis
AKI	Acute kidney injury
AKIN	Kidney injury network
Anti-PLA2R	Anti-phospholipase A2 receptor
BMI	Body mass index
CG	Cockcroft–Gault
CKD	Chronic kidney disease
CKD-EPI	Chronic Kidney Disease Epidemiology Collaboration
CTLA	Cytotoxic T-lymphocyte-associated protein
DMSO	Dimethyl sulfoxide
eCrC	Estimated creatinine clearance
e-GFR	Estimated-Glomerular filtration rate
FDA	Food and drug administration
GFR	Glomerular filtration rate
GVHD	Graft versus host disease
HSCT	Hematopoietic stem-cell transplantation
ICPIs	Immune checkpoint inhibitors
IV	Intravenous
KDIGO	Kidney Disease: Improving Global Outcome
KDOQI	Kidney Foundation—Kidney Disease Outcome Quality Initiative
MGRS	Monoclonal gammopathy of renal significance
MIS	Marrow infusion syndrome
MM	Multiple myeloma
MN	Membranous nephropathy
MPG	Membranoproliferative glomerulonephritis
NCCN	National Comprehensive Cancer Network
NSAIDs	Non-steroidal anti-inflammatory drugs
OCT	Organic cation transporter
PD	Programmed death
PGNMID	Proliferative glomerulonephritis with monoclonal immunoglobulin deposits
RAAS	Renin–angiotensin–aldosterone system
RBCs	Red blood cells
RCC	Renal cell carcinoma
RI	Renal insufficiency
RIFLE	Risk, Injury, Failure, Loss and End-Stage Kidney
RPG	Glomerulonephritis
sCr	Serum creatinine
SIOG	Society of Geriatric Oncology
THSDA7A	Anti-thrombospondin type-1 domain-containing 7A
TLS	Tumor lysis syndrome
TMA	Thrombotic microangiopathy
VEGF	Vascular endothelial grow factor
